# Inflammation and damage-associated molecular patterns in major psychiatric disorders

**DOI:** 10.47626/2237-6089-2022-0576

**Published:** 2023-10-31

**Authors:** Fernanda Endler Valiati, Jacson Gabriel Feiten, Luiza Paul Géa, Érico de Moura Silveira, Ellen Scotton, Marco Antonio Caldieraro, Giovanni Abrahão Salum, Marcia Kauer-Sant’Anna

**Affiliations:** 1 Laboratório de Psiquiatria Molecular Centro de Pesquisa Clínica Hospital de Clínicas de Porto Alegre Porto Alegre RS Brazil Laboratório de Psiquiatria Molecular, Centro de Pesquisa Experimental (CPE) and Centro de Pesquisa Clínica (CPC), Hospital de Clínicas de Porto Alegre (HCPA), Porto Alegre, RS, Brazil.; 2 Departamento de Bioquímica Instituto de Ciências Básicas da Saúde Universidade Federal do Rio Grande do Sul Porto Alegre RS Brazil Departamento de Bioquímica, Instituto de Ciências Básicas da Saúde, Universidade Federal do Rio Grande do Sul (UFRGS), Porto Alegre, RS, Brazil.; 3 Departamento de Psiquiatria Faculdade de Medicina UFRGS Porto Alegre RS Brazil Programa de Pós-Graduação em Psiquiatria e Ciências do Comportamento, Departamento de Psiquiatria, Faculdade de Medicina, UFRGS, Porto Alegre, RS, Brazil.; 4 Instituto Nacional de Ciência e Tecnologia Translacional em Medicina Porto Alegre RS Brazil Instituto Nacional de Ciência e Tecnologia Translacional em Medicina (INCT-TM), Porto Alegre, RS, Brazil.; 5 Programa de Pós-Graduação em Farmacologia e Terapêutica UFRGS Porto Alegre RS Brazil Programa de Pós-Graduação em Farmacologia e Terapêutica, UFRGS, Porto Alegre, RS, Brazil.

**Keywords:** Psychiatric disorders, inflammation, DAMPs, HSP70, psychotropic medication

## Abstract

**Background:**

Emerging evidence indicates that inflammation plays an important role as a mechanism underlying mental disorders. However, most of the research on inflammatory mechanisms focuses on serum levels of interleukins and very few studies have investigated molecules that initiate and expand innate immune pathways such as damage-associated molecular patterns (DAMPs).

**Objectives:**

This study investigated the levels of DAMPs among patients diagnosed with major depressive disorder (MDD), bipolar disorder (BD) I and II, schizophrenia (SCZ), and generalized anxiety disorder (GAD). We quantified serum levels of heat shock proteins (HSPs) 70 and 60 and of S100 calcium-binding protein B (S100B).

**Methods:**

Serum levels of HSP70, HSP60, and S100B were assessed in a sample of participants with psychiatric disorders (n = 191) and a control group (CT) (n = 59) using enzyme-linked immunosorbent assay (ELISA).

**Results:**

Serum HSP70 concentrations were significantly higher in the MDD group compared to the CT, SCZ, and BD groups. The GAD group had higher concentrations of HSP70 than the SCZ group. Exploring associations with medications, lithium (p = 0.003) and clozapine (p = 0.028) were associated with lower HSP70 levels. Approximately 64% of the sample had DAMPs levels below the limits of detection indicated by the respective ELISA kit.

**Conclusion:**

This was the first study to assess DAMPs levels in a transdiagnostic sample. Our preliminary findings suggest that HSP70 may be associated with MDD pathophysiology. Medications such as lithium and clozapine were associated with lower HSP70 levels in BD and SCZ groups, respectively. Therefore, it is worth mentioning that all participants were medicated and many psychotropic drugs exert an anti-inflammatory effect, possibly reducing the signs of inflammation.

## Introduction

Mental disorders are characterized by disturbance in cognition, emotional regulation, or behavior that reflects a dysfunction in the psychological, biological, or developmental processes.^1,2^ They are responsible for major medical, societal, and economic burdens and are one of the main causes of disability worldwide.^3^ Preclinical and clinical evidence has demonstrated a strong association between the pathophysiology, progression, and symptom severity of psychiatric disorders and inflammation (for a review see Jeppesen and Benros^4^ and Felger^5^), in a bidirectional relationship.^6^ Understanding the mechanisms by which inflammation is associated with psychiatric disorders might help the development of new treatments and testing of existing drugs with known mechanisms (drug repurposing).

The biological response to stress is associated with both central (i.e., neuroinflammation) and peripheral inflammatory responses and their biochemical cascades including inflammasome, mitogen-activated protein kinase (MAPK), and nuclear factor (NF)-κB pathways.^7^ Patients with major mental illness exhibit all cardinal features of inflammation, including increased circulating levels of inflammatory inducers, activated sensors expressed on immune and nonimmune cells, inflammatory mediators, and target tissues that are modulated by them.^6^ Indeed, mounting evidence suggests that stress-induced production of inflammatory factors such as cytokines, chemokines, prostaglandins, and reactive oxygen species promotes pathogenic effects in psychiatric disturbances.^6,8^ However, these stress-induced changes vary from one individual to another with some subjects being vulnerable and others resilient.^9^

It has long been postulated that acute and chronic stress play a significant role in the onset of severe and impairing psychiatric conditions, exacerbation of symptoms, and pathogenesis of psychiatric disorders.^9-11^ Previously, our group has suggested that “allostatic load,” a concept from general health studies, also plays a role in the field of psychiatry, particularly with relation to bipolar disorder (BD).^12^ Briefly, this concept relates the neural and bodily “wear and tear” associated with sustained exposure to chronic stress to psychopathology. This body-brain link would partially explain the enhanced vulnerability to stress, cognitive impairment, and higher rates of physical comorbidity and mortality in psychiatric disorders.^12^ However, the common pathophysiology of psychiatry disorders and the exact mechanisms by which inflammation is initiated and propagated remain unclear.

Emerging evidence indicates an important role of sterile inflammation, in particular of inflammation involving molecules that initiate and expand innate immune pathways.^6,13^ Some of these molecules are known as DAMPs, including heat shock proteins (HSPs) and S100 family proteins.^14^ DAMPs are endogenous signals released during cell stress, damage, or death, and could act as triggers for the immune activation and systemic toxicity observed in psychiatry.^7^ A previous study by our group was the first to show increased DAMPs levels in the serum of patients with BD, providing a link between BD, immune activation, and systemic toxicity.^15^ DAMPs constitute a heterogeneous group of molecules that originate either from different cell compartments or from the extracellular space.^16^ This vast group of danger signals has also been reported in patients with major depressive disorder (MDD), schizophrenia (SCZ), and generalized anxiety disorder (GAD).^17-21^

Identification and functional characterization of DAMPs and the associated inflammatory pathways in psychiatric disorders can be insightful. Both HSPs and S100 proteins can stimulate production of pro-inflammatory cytokines in vitro and play roles in inducing sterile inflammation in vivo.^14^ Thus, here we aimed to quantify serum levels of a defined set of DAMPs (specifically, HSP70, HSP60, and S100B), replicating our previous findings in BD^15^ in a transdiagnostic sample, including individuals with MDD, BD I and II, SCZ, and GAD and a control group (CT). Such a transdiagnostic approach has been recommended as a strategy for investigating the pathophysiological basis in psychiatry, focusing also on non-specific mechanisms or domain-specific findings, rather than limiting investigation to diagnosis-specific studies. This approach may provide a new perspective on the relationship between inflammation and psychiatric disorders.^17,18^

## Methods

### Subjects

Patients were recruited from March 2015 to June 2016 and healthy subjects were enrolled from February to June 2019. All patients recruited were already stable and on regular psychotropic medication. Individuals with MDD (n = 41), BD types I and II (n = 58), SCZ (n = 53), and GAD (n = 39) were recruited at depressive disorders, bipolar disorders, schizophrenia, and anxiety disorders outpatient clinics, respectively, at the Hospital de Clínicas de Porto Alegre (HCPA), state of Rio Grande do Sul, Brazil. All patients underwent a comprehensive clinical interview with a psychiatrist, and diagnoses were made using Diagnostic and Statistical Manual of Mental Disorders, 4th edition, Text Revision (DSM-IV-TR) criteria (described below).

The inclusion criterion was a diagnosis of MDD, BD, SCZ, or GAD according to the DSM-IV-TR criteria.^19^ Exclusion criteria comprised current abuse of alcohol or illicit substances, presence of metabolic disorders (such as diabetes), chronic inflammatory diseases (e.g., inflammatory bowel disease or rheumatoid arthritis), cancer, other severe medical conditions, pregnancy and breastfeeding, and organic mental illness.

A healthy comparison group (n = 59) was recruited from the blood bank of the Hemotherapy Service at the same hospital. Subjects were screened using a questionnaire and had no history of psychiatric illness. The questionnaire screened for the following criteria: not having a history of any mental disorders; never having attempted suicide; no psychiatric hospital admissions; never using psychiatric medication; and never having used drugs of abuse or psychoactive substances. The exclusion criteria for controls were the same used for cases.

The study design was approved by the Research Ethics Committee at the HCPA (protocols 14-0515 and 18-0114). All participants provided written informed consent (which was approved by the local ethics committee) after the nature of the procedures had been fully explained to them.

### Assessment

Sociodemographic and clinical data were collected in an extensive interview session conducted with the participants. All patients were evaluated using the same protocol. Sociodemographic status was evaluated using the 2015 Economic Classification Criterion Brazil.^20^ The diagnostic assessment was systematized by application of an Electronic Chart Review Instrument (ECRI) for each participant.^21,22^ Three trained psychiatrists used the instrument to confirm or refute the primary DSM-IV diagnosis for each participant. This instrument ensures a standardized method to collect information from electronic charts and has the advantage of offering a longitudinal view of each person’s psychiatric history based on an electronic chart review guided by checking each set of DSM-IV-TR criteria. Age at onset of first symptoms, suicide attempts, and number of psychiatric hospitalizations were also recorded/collected as part of the chart review. Current dimensional mood, anxiety symptoms, severity of illness, and functionality were assessed using the Hamilton Depression Rating Scale (HDRS),^23,24^ the Young Mania Rating Scale (YMRS),^25,26^ the Generalized Anxiety Disorder 7 (GAD-7),^27-29^ and the Functioning Assessment Short Test (FAST),^30^ respectively.

### Blood samples

Blood samples were collected by venipuncture from all patients and controls and allowed to clot in blood collection tubes with no additives. Subsequently, the whole blood was centrifuged for 10 minutes at 1,000 × *g* and room temperature, and the serum was removed, aliquoted, and stored at -80 °C until assayed.

### Biochemical assays

All assays were performed with serum samples using colorimetric enzyme-linked immunosorbent assay (ELISA) kits according to the manufacturer’s instructions with minor modifications (HSP70 Catalog #DY1663, HSP60 Catalog #DYC1800, and S100B Catalog #DY1820; R&D Systems, Inc., MN, USA). Samples were not diluted and values were expressed as pg/mL.

### Statistical analysis

Statistical analysis was performed using R software, version 4.0.2 (R Core Team, 2019). The normal distribution of dependent variables was evaluated using the Shapiro-Wilk test. If assumptions were met, DAMPs levels were compared between groups using the multiple groups Kruskal-Wallis H test followed by Dunn’s test. Importantly, a 0 value was adopted for HSP70, HSP60, and S100B levels that were below the limit of detection indicated in each ELISA kit. Chi-square tests were used to evaluate associations between categorical variables.

**Figure 1:**
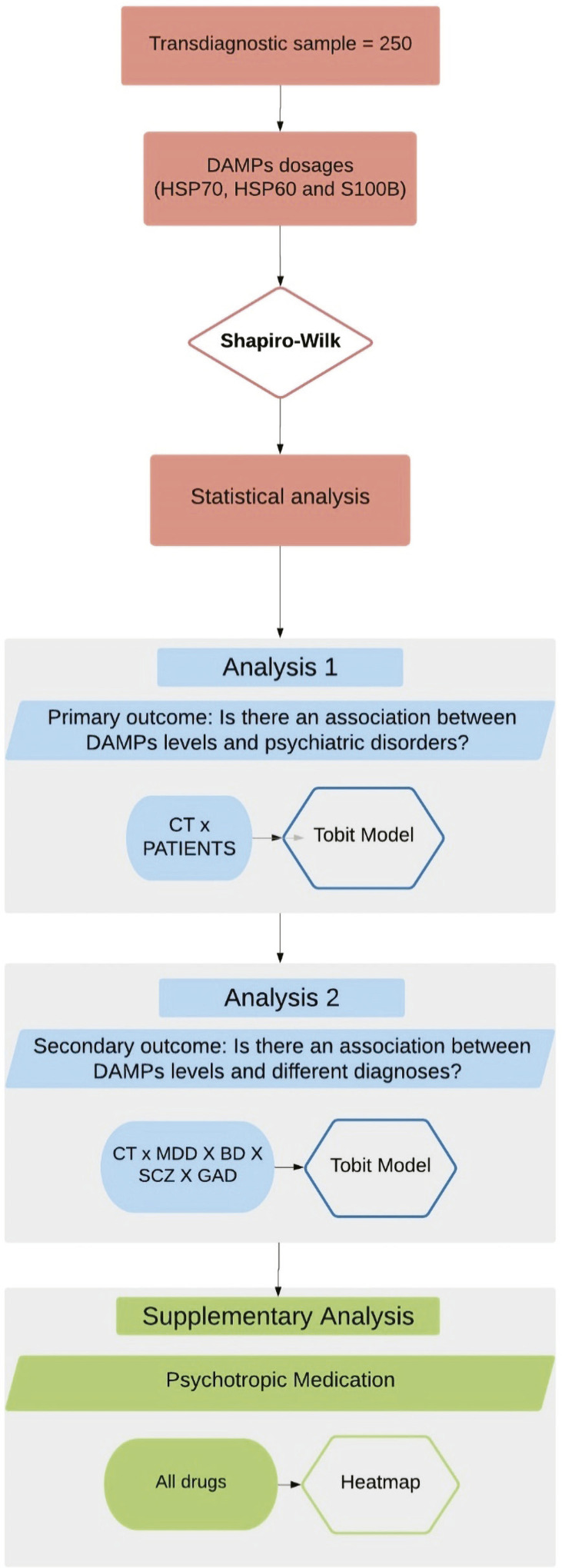
Flowchart illustrating the stepwise statistical analysis performed. BD = bipolar disorder; CT = control; DAMPs = damage-associated molecular patterns; GAD = generalized anxiety disorder; HSP = heat shock proteins; MDD = major depressive disorder; SCZ = schizophrenia.

Due to a highly skewed distribution (i.e., few detected samples), the concentration of HSP70 was analyzed using the tobit regression model. This was performed using the VGAM for R package version 1.1.3 to include variables that might confound the results. This model is designed to estimate linear relationships between variables when there are partially unknown values, since, in our case, values below the limit of detection were censored (https://stats.idre.ucla.edu/r/dae/tobit-models/). The tobit model creates a latent variable for modeling the censored data and the coefficients refer to the latent variable.^31^ The strategies employed for statistical analysis of the results are illustrated in a flowchart shown in Figure 1.

## Results

### Sample characteristics

Overall, the majority of participants were women (57.6%), who had secondary education (50.4%). Ages ranged from 18 to 60 years (42.8 ± 12.5 years). Patients had a long course of illness (mean ± standard deviation, 16.4 ± 12.7 years). Table 1 summarizes the sociodemographic and clinical characteristics of the sample by diagnostic groups.

**Table 1:** Sociodemographic and clinical characteristics of participants

	CT (n = 59)	MDD (n = 41)	BD (n= 58)	SCZ (n = 53)	GAD (n = 39)
Age at enrollment (years)^1^*	39.0 ± 11.0^a^	50.5 ± 11.1^a,b,c^	45.1 ± 13.4	38.6 ± 10.4^b^	42.7 ± 13.2^c^
Female sex^2^*	23 (39)^a^	36 (87)^b^	43 (74)^c^	14 (26)^d^	28 (71)
Education^2^*					
Primary	6 (10.2)^a^	21 (51.2)^c^	18 (31.0)	19 (35.8)	16 (41.0)
Secondary	32 (54.2)	16 (39.0)	31 (53.4)	29 (53.4)	18 (46.2)
Tertiary	21 (35.6)^b^	4 (9.8)	9 (15.5)	5 (9.4)	5 (12.8)
Suicide attempters^2†^	-	20 (48.8)	33 (56.9)^a^	17 (32.1)	9 (23.1)^b^
Number of suicide attempts^1^*	-	1.0 ± 1.5 0 (0-2)^a^	1.5 ± 1.9 1 (0-2)^b,c^	0.58 ± 1.0 0 (0-1)^b^	0.38 ± 0.9 0 (0-0)^a^
Number of hospitalizations^1^*	-	0.54 ± 1.1^a,b^	3.1 ± 3.1^a,d^	3.2 ± 2.8^b,c^	0.7 ± 1.3^c,d^
Disorder duration (years)^3^	-	13 (8-24)	16 (11-28)	14 (8-23)	10 (6-24.5)
YMRS^1^	-	1.6 ± 2.0	2.1 ± 2.7	1.9 ± 3.8	1.2 ± 1.6
HDRS^1^*	-	12.7 ± 6.5^a^	10.0 ± 6.0 ^c^	5.2 ± 4.0^a,b,c^	11.2 ± 7.2^b^
GAD-7*		19.9 ± 5.9^a,b,c^	16.3 ± 6.1^c,d^	12.4 ± 4.7^a,d,e^	16.6 ± 6.6^b,e^
FAST*	-	30.4 ± 12.2^a,b^	31.8 ± 15.8^c^	24.3 ± 15.0^a,c^	26.0 ± 14.0^b^

BD = bipolar disorder; CT = control; FAST = Functional Assessment Short Test; GAD 7 = Generalized Anxiety Disorder Scale 7; GAD = generalized anxiety disorder; HDRS = Hamilton Depression Rating Scale; MDD = major depressive disorder; SCZ = schizophrenia; YMRS = Young Mania Rating Scale.

Letters a-e indicate groups with statistical differences.

^1^ Values are expressed as mean (standard deviation); ^2^ Values are expressed as n (percentage); ^3^ Data are expressed as median and interquartile range (IQR).

* p < 0.001 and ^†^ p < 0.01 indicate significance levels in ANOVA, Kruskal-Wallis, and 𝜒-squared tests.

### Serum levels of HSP70, HSP60, and S100B

**Figure 2:**
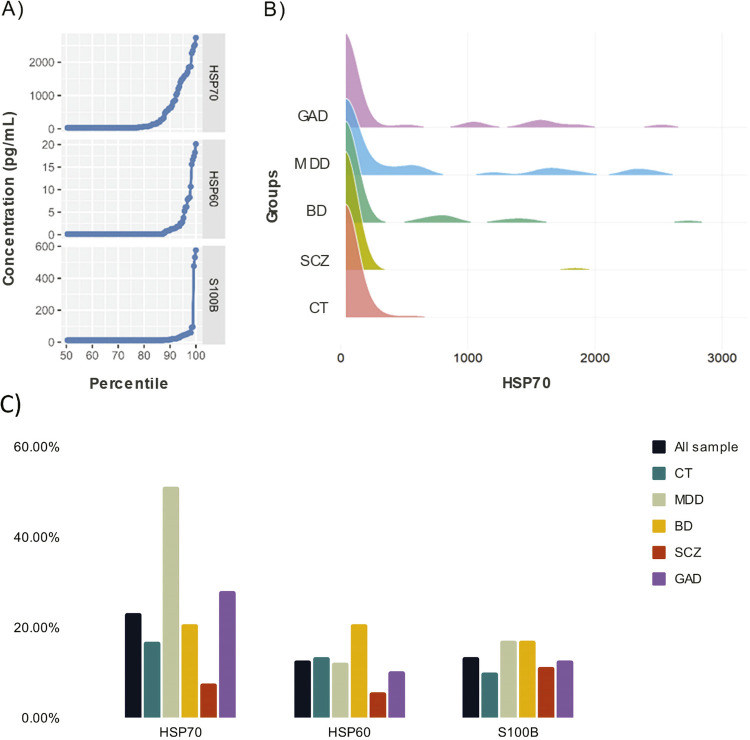
Damage-associated molecular patterns (DAMPs) assay data. A) Ordered values of DAMPs concentrations. B) Distribution of concentration of heat shock proteins (HSP) 70 in each group. C) Detection (%) of HSP70, HSP60, and S100B in the whole sample and in each group. BD = bipolar disorder; CT = control; GAD = generalized anxiety disorder; MDD = major depressive disorder; SCZ = schizophrenia.

Figure 2 shows the DAMPs assay data. The concentrations of HSP70, HSP60, and S100B within the 95th percentile were 1,630, 5.7, and 46.1 pg/mL, respectively (Figure 2A). The HSP70 concentration in each group is shown in Figure 2B. Approximately 62% of the sample had HSP70, HSP60, or S100B levels below the limits of detection indicated in the respective ELISA kit (Figure 2C). For HSP70, 58 participants had detectable levels (control, n = 10; MDD, n = 21; BD, n = 12; SCZ, n = 4; GAD, n = 11). HSP60 levels were detected in only 32 participants (control, n = 8; MDD, n = 5; BD, n = 12; SCZ, n = 3; GAD, n = 4), and S100B was detected in 34 participants (control, n = 6; MDD, n = 7; BD, n = 10; SCZ, n = 6; GAD, n = 5).

### High serum levels of HSP70 in MDD

**Figure 3:**
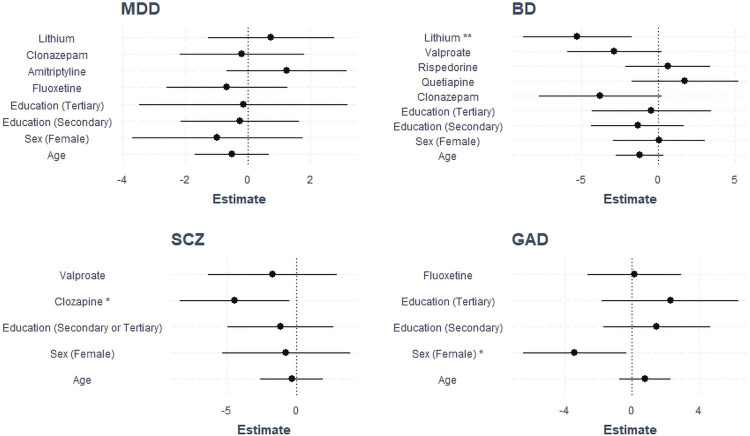
Coefficient confidence intervals for tobit models are based on major depressive disorder (MDD), bipolar disorder (BD), schizophrenia (SCZ), and generalized anxiety disorder (GAD). Bars represent 95% confidence intervals. * p < 0.05; ** p < 0.01.

No statistical differences in HSP70 (p = 0.097), HSP60 (p = 0.989), or S100b (p = 0.353) levels were found between the transdiagnostic group (which included all patients with MDD, BD, SCZ, or GAD) and the CT. When the groups of subjects with psychiatric disorders (i.e., MDD, BD, SCZ, and GAD) were separated, our analysis showed that HSP70 was significantly different between the different diagnostic groups and CT. Interestingly, tobit analysis indicated that HSP70 levels were higher in MDD than in the CT (p < 0.001), SCZ (p < 0.001), and BD (p = 0.002) groups. Moreover, the GAD group had higher HSP70 concentrations than the SCZ group (p = 0.002) (see Figure 2B for the distributions in each group and Table 2 for statistical results of HSP70 levels in the sample). Similar results for HSP70 levels were found after controlling for age, sex, education, and psychiatric medication (Figure 3). No differences were found in HSP60 or S100B serum levels between groups.

**Table 2:** Multiple comparisons for HSP70 levels

Comparison	Estimate	Adj. p-value
MDD-SCZ	5.17	< 0.001*
MDD-CT	3.67	< 0.001*
MDD-BD	2.51	0.006^†^
GAD-SCZ	3.33	0.006^†^
BD-SCZ	2.66	0.021^‡^
MDD-GAD	1.84	0.062
GAD-CT	1.82	0.078
CT-SCZ	1.51	0.185
BD-CT	1.15	0.218
GAD-BD	0.67	0.436

BD = bipolar disorder; CT = control; GAD = generalized anxiety disorder; HSP = heat shock proteins; MDD = major depressive disorder; SCZ = schizophrenia.

p-values were adjusted with the Benjamini-Hochberg method.

Tobit test. * < 0.001; ^†^ < 0.01; ^‡^ < 0.05.

### Influence of medication on serum HSP70 levels

All patients in the study were on continuous use of psychotropic medication. Table 3 shows the drugs most commonly used by patients in each group. Valproate, clonazepam, lithium, fluoxetine, and risperidone were used by at least one patient in all groups. Fluoxetine was the drug most used by MDD and GAD patients (i.e., 63.41% and 53.85%, respectively). The most frequent medications for patients with BD I and II were valproate (53.45%) and lithium (41.38%). Furthermore, the SCZ group was treated with clozapine (73.58%) and valproate (24.53%). The data on these and other psychotropic drugs can be found in Table 3 and are illustrated in Figure S1, which is available as online-only supplementary material.

**Table 3:** Psychotropic drugs most used by MDD, BD, SCZ, and GAD patients

Psychotropic drug, n (%)	MDD (n = 41)	BD (n = 58)	SCZ (n = 53)	GAD (n = 39)	p-value*	p-value^†^
Fluoxetine	26 (63.0)	7 (12.0)	7 (13.0)	21 (54.0)	< 0.001	< 0.001
Amitriptyline	14 (34.0)	0 (0.0)	8 (15.0)	4 (10.0)	< 0.001	< 0.001
Clonazepam	13 (32.0)	13 (22.0)	10 (19.0)	4 (10.0)	0.12	0.2
Lithium	9 (22.0)	24 (41.0)	4 (7.5)	1 (2.6)	< 0.001	< 0.001
Diazepam	5 (12.0)	1 (1.7)	2 (3.8)	2 (5.1)	0.2	0.2
Valproate	3 (7.3)	31 (53.0)	13 (25.0)	3 (7.7)	< 0.001	< 0.001
Risperidone	4 (9.8)	21 (36.0)	9 (17.0)	4 (10.0)	0.002	0.004
Quetiapine	0 (0.0)	12 (21.0)	0 (0.0)	2 (5.1)	< 0.001	< 0.001
Clozapine	1 (2.4)	8 (14.0)	39 (74.0)	0 (0.0)	< 0.001	< 0.001
Haloperidol	4 (9.8)	1 (1.7)	4 (7.5)	5 (13.0)	0.14	0.2
Paroxetine	1 (2.4)	2 (3.4)	0 (0.0)	4 (10.0)	0.066	0.11
Sertraline	4 (9.8)	4 (6.9)	2 (3.8)	4 (10.0)	0.6	0.6
Others	16 (39.0)	27 (47.0)	15 (28.0)	14 (36.0)	0.3	0.3

BD = bipolar disorder; GAD = generalized anxiety disorder; MDD = major depressive disorder; SCZ = schizophrenia

* Pearson’s chi-square test, Fisher’s exact test; ^†^ False discovery rate corrected for multiple testing.

The tobit regression model showed a significant association between treatment and serum HSP70 levels in BD and SCZ (Figure 3). We only included psychotropic drugs used by at least 20% of the patients in each group in the tobit models. The associations were adjusted for age, gender and education level. Specifically, decreased HSP70 levels were observed in BD patients on treatment with lithium (p = 0.003). Interestingly, serum levels of HSP70 were detected in only one of the 39 patients who were taking clozapine. This result suggests that clozapine may be associated with lower levels of DAMPs in SCZ (p = 0.028).

## Discussion

This is the first study in the field of psychiatry to evaluate the influence of DAMPs, including HSP70, HSP60, and S100B, in a transdiagnostic sample. Our findings showed that MDD is associated with higher serum HSP70 levels, particularly in comparison to CT and SCZ. Furthermore, the use of psychotropic medication such as lithium and clozapine might be associated with lower serum HSP70 levels in BD and SCZ patients, respectively. Despite the relevance of our findings, it is worth mentioning that approximately 64% of the sample had undetectable serum biomarker levels. This is described further as part of the discussion of the study’s limitations.

There is abundant evidence showing that a significant proportion of patients with mood, psychotic, and anxiety-related disorders exhibit upregulation of inflammatory markers such as cytokines, chemokines, and acute phase reactants at the gene and protein level, and increased presence of pro-inflammatory immune cell phenotypes in the blood and cerebrospinal fluid.^4,5,32^ This increased inflammatory state has also been associated with an elevated risk of development and symptom severity of these disorders.^32,33^ It has been extensively reported that a single major life event or an acute intense stressor exposure can also increase inflammatory markers, such as DAMPs, by triggering a detectable local and systemic sterile inflammatory response.^33-35^ DAMPs are altered metabolism products of necrotic or stressed cells, which are deemed to be alarm signals by the innate immune system.^14,36^ They represent a heterogeneous group of molecules originating in either intracellular or extracellular space.^16^

Various intracellular DAMPs are released from the mitochondria, the autophagosome, the endoplasmic reticulum, the nucleus, and the cytosol.^7,16^ A variety of stressful situations, including environmental, pathological, or physiological stimuli, induce a marked increase in synthesis of HSPs, which leak into the extracellular compartment and activate immune signaling pathways.^7,37^ HSPs are considered tissue alarmins and can also act as DAMPs by interacting with toll-like receptors (TLRs).^19,20^ These molecules trigger innate immune responses by inducing release of cytokines and chemokines and activating natural killer cells.^19^ Likewise, extracellular S100 proteins influence innate and adaptive immune responses by their wide-ranging regulatory actions on macrophages, monocytes, neutrophils, lymphocytes, microglia, mast cells, and vascular smooth muscle cells.^7,38^ These proteins have been considered markers of brain injury in several pathologies.^21^ Our study evaluated HSP70, HSP60, and S100B serum levels among different psychiatric disorders. Only HSP70 was significantly different among groups and, specifically, serum levels were higher in MDD. In general, DAMPs are present in the circulation under normal physiological conditions, and their circulating levels seem to decrease with age and increase in a number of pathological conditions.^37,39,40^ However, the mechanisms and physiological significance of release of HSPs and S100 proteins are still unclear.^7,37^

Compelling clinical research has established that repeated, chronic, and excessive life or experimental laboratory stressor exposure, involving social conflict, threat, isolation, and rejection, increases inflammatory states (for reviews see Heil and Land,^39^ Kiecolt-Glasera et al.,^41^ Suzanne and Segerstrom,^42^ and Slavich and Irwin^43^). Inflammation plays an important role in the pathophysiology of depression. Although the clinical evidence for HSP70 DAMP activity in psychiatric disorders is limited, several studies have reported increased HSP70 serum levels associated with depression (for a review, see Franklin et al.^44^). Our study showed higher HSP70 levels in MDD, compared to both healthy subjects and patient groups (i.e., SCZ and BD). Among HSPs, which mediate inflammatory responses to stress, HSP70 has been implicated in different mental disorders, particularly in MDD.^44-46^ Several preclinical studies have reported significant changes in expression of HSP70 in animal models of depression.^33,47,48^ Likewise, a number of clinical studies have described an increase in serum HSP70 levels in depressed patients or people at-risk of developing MDD.^45,46,49^ In addition, animal models of chronic stress showed that depressive-like behaviors coincide with increased levels of HSP70 in the hippocampus and prefrontal cortex.^47,48,50,51^ Similarly, an increase in central and peripheral levels of HSP70 has been reported following acute exposure to stress.^8,50,52^ Altogether, these findings constitute strong evidence for the association between depression and inflammation related to DAMP HSP70, revealing a link between MDD, sterile immune activation, and systemic toxicity.

Previously, our group assessed DAMPs levels in drug-free patients with BD during mood episodes. For the first time, an increase in serum levels of HSP70 was demonstrated in these patients. However, no difference was observed in HSP60 levels compared to controls.^15^ In the present study, we investigated the role of DAMPs in a transdiagnostic sample and, in contrast to the previous study, all patients recruited were already stable and on regular treatment with psychotropic medication, which may explain in part why we did not detect differences between BD and controls. In fact, most of the BD patients were on lithium, which was associated with lower HSP70 levels. Although no differences were observed in HSP60 or S100B serum levels between groups, HSP70 levels were significantly higher in MDD compared to CT and SCZ. Moreover, a significant association was observed between psychotropic medication and HSP70 serum levels in BD and SCZ. The findings of the present study, combined with our previous results in BD may indicate that HSP70 is chronically elevated in MDD but decreases to levels similar to those of healthy controls in BD and SCZ patients presenting with remission of symptoms. However, longitudinal assessments would be necessary to confirm this hypothesis.

A set of targets related to oxidative stress, neurotrophins, and inflammation as key mediators of the cognitive and general health impairments that occur with recurrent mood episodes were reported for the first time en bloc on a systemic toxicity index (STI).^53^ The same STI was evaluated in early-stage mood disorders and use of medications was associated with significantly lower toxicity.^54^ In the present study, the mood stabilizer lithium and the antipsychotic clozapine were associated with lower levels of HSP70 in BD and SCZ groups, respectively. This is in line with a number of recent reviews and meta-analyses suggesting that most of psychotropic medications have anti-inflammatory effects, which may play a role in the treatment of mood symptoms and psychosis.^5,55,56^ Several hypotheses have been suggested to explain its mechanism of action, signaling molecules, cellular pathways, and pharmacological effects.^32,57-64^ It is worth noting that the profile of medications used in our sample reflects the government treatment policy. Therefore, preferred medications are those provided free of charge on public health protocols. In addition, the high prevalence of clozapine use is associated with the fact that our hospital runs a referral Clozapine Clinic, which should be taken into account when interpreting the results.

It has been established that stress induces sterile inflammation in psychiatric disorders. Peripheral and central innate immune systems become activated in response to DAMPs.^14^ These factors bind to pattern recognition receptors (PRR) primarily on innate immune cells and promote proinflammatory cytokine production through activation of NF-κB and the NLRP3 inflammasome complex.^44^ In turn, the proinflammatory signaling enhances activation of inflammatory cascades, resulting in positive feedback and further stimulation of inflammatory signaling.^44^ Our results suggest that drugs are possibly mediating this inflammation status. It is known that lithium inhibits glycogen synthase kinase (GSK)-3β, resulting in decreased NF-κB activity. Thus, the reduction in NF-κB activity through translocation of p65-p50 to the nucleus and stimulation of its transcriptional activity leads to attenuated expression and decreases production of inflammatory-associated mediators and enzymes (for a review, see Ramirez et al.^63^). Also, an antipsychotic anti-inflammatory action seems to be associated with NF-κB activity as well. Upregulation of MAPK, protein kinase (PK)-C pathway, and elevation of intracellular Ca2+ concentration occurs by inflammatory transactivations evoked by interferon (IFN)-γ and/or lipopolysaccharide (LPS). These intracellular activations translocate NF-κB from cytosol to nucleus and produce inflammatory cytokines and nitric oxide via nucleus response. Antipsychotics seem to have potential inhibitory effects by modulating these intracellular cascades, such as MAPK, PKC pathway, calcium signaling, and NF-κB, reducing inflammatory molecule release (for a review, see Husain et al.^60^). Thus, these medications could possibly be inhibiting the NF-κB pathway and preventing inflammation.

There is a large subset of patients with MDD who do not exhibit adequate response to current antidepressant therapies; hence, these patients are generally characterized as having treatment-resistant depression.^65^ Moreover, the effects of selective serotonin reuptake inhibitors (SSRIs), such as fluoxetine, on inflammatory response are still contradictory as studies report these drugs as either anti-inflammatory or proinflammatory agents.^61^ In this regard, evidence of both effective responses to anti-inflammatory drugs and dysregulated inflammatory signaling in treatment-resistant MDD underscores the importance of further investigating the mechanisms by which sterile inflammation contributes to the onset and course of depression.^65,66^ However, the link between psychiatric disorders and inflammation appears to be bidirectional, and simply reducing DAMPs only is not a sustainable solution. Instead, the inflammatory agents leading to their generation ought to be recognized and avoided, since in some cases, drugs taken to alleviate some DAMPs can end up generating other DAMPs.^7^

The present study has some limitations. First, we had a low rate of detection of serum DAMPs levels across all groups. The sensitivity of the kit used in the present study is three times lower than the kit used in the previous study performed by our research group.^15^ Second, the scores of the scales for dimensional mood and anxiety symptoms, severity of illness, and functionality were not used in the data analysis. Third, all patients were taking at least one medication and were stable, without any clinical symptoms at the time of sample collection. Fourth, it is possible that the combination of the two previous factors led to lower detection of the DAMPs tested, impairing more robust data analyses. Fifth, medications were included in the model without taking into account the effects of combination therapy, which warrants caution when considering the results for any single drug. However, the high rate of detection of HSP70 levels in the MDD allowed us to identify a significant association. Further studies are needed to establish possible major connections between different psychiatric disorders and DAMPs levels. Overall, we envisage that better characterization of release of DAMPs and their immunomodulatory properties from a transdiagnostic perspective will provide new insights into their immune-regulatory role in the psychiatric field and, possibly, into their use as prognostic biomarkers that could improve the way we classify and treat mental disorders.

## Conclusion

Mounting evidence has demonstrated a strong association between depression and expression of factors that increase inflammation. Our findings indicate that inflammatory signaling including levels of HSP70 was associated with MDD. Increases in extracellular levels of DAMPs, such as HSP70, appear to trigger sterile inflammatory signaling following physical and psychological stress via NF-κB activity. Also, the sample in this study was being treated with psychotropic medications. This proved relevant, since use of some medications was associated with lower levels of HSP70. Transdiagnostic research aims to provide novel insights into how we might understand mental health difficulties since psychiatric disorders share common etiological and maintenance processes as well as cognitive-affective, interpersonal, and behavioral features. Removing the distinctions between proposed psychiatric taxa at the level of classification and the identification of key DAMPs opens up new ways of thinking about the onset, maintenance, and clinical treatment of and recovery from experiences of disabling mental distress. Future studies should also consider exploring other DAMPs in transdiagnostic samples, to bridge the understanding of peripheral and central interactions, contributing to the etiology and pathophysiology of these disorders.
