# Supplementary material for: Inflammation and damage-associated molecular patterns in major psychiatric disorders
Source: Trends Psychiatry Psychother. 2023 Oct 31;45:e20220576. doi: 10.47626/2237-6089-2022-0576 (PMC10640887; doi:10.47626/2237-6089-2022-0576)
Supplement: Supplementary file 1 [file 2238-0019-trends-45-e20220576-suppl.pdf]

**Trends in Psychiatry and Psychotherapy - Online-Only Supplementary Material**
http://doi.org/10.47626/2237-6089-2022-0576
Inflammation and damage-associated molecular patterns in major psychiatric disorders - Valiati et al.

**Figure S1** - Data for all psychotropic drugs used by patients.

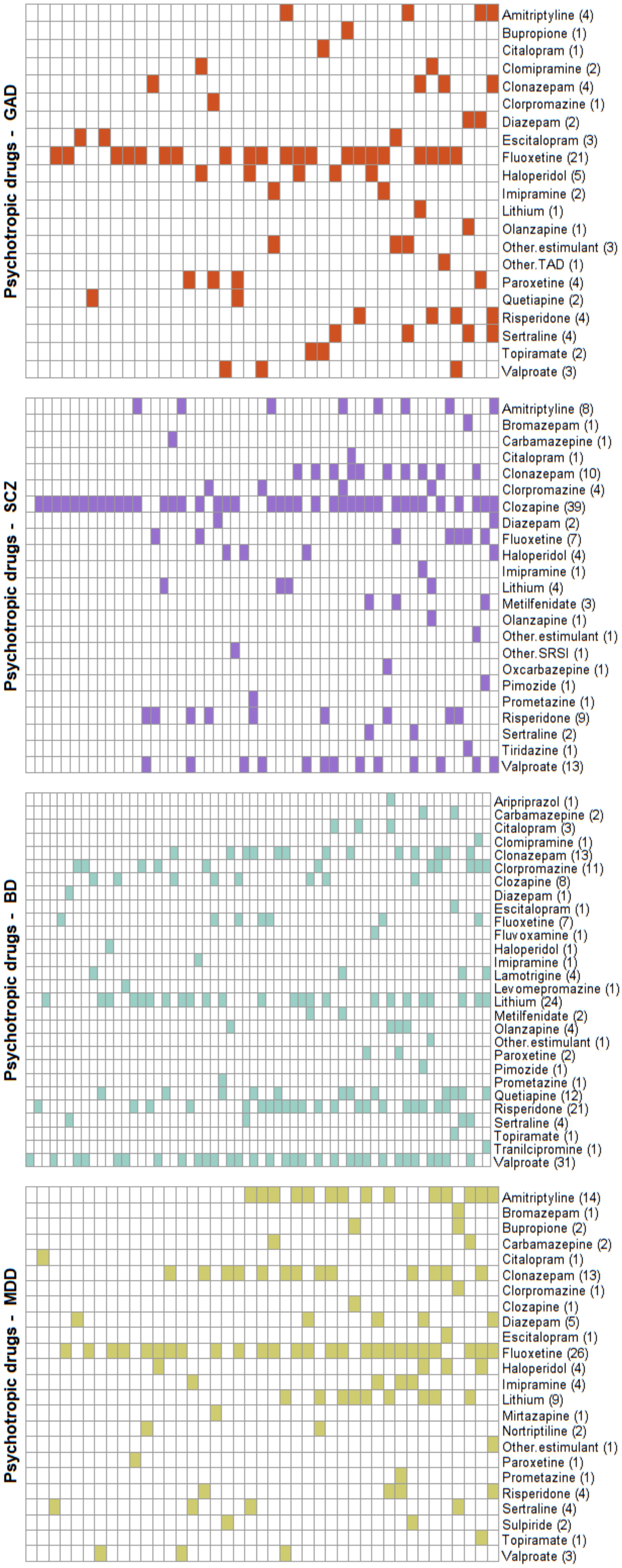


BD = bipolar disorder; GAD = generalized anxiety disorder; MDD = major depressive disorder; SCZ = schizophrenia.
